# Evolution of the Macrophage CD163 Phenotype and Cytokine Profiles in a Human Model of Resolving Inflammation

**DOI:** 10.1155/2013/780502

**Published:** 2013-05-02

**Authors:** Betsy J. Evans, Dorian O. Haskard, Gregory Sempowksi, R. Clive Landis

**Affiliations:** ^1^Eric Bywaters Centre for Vascular Inflammation, Faculty of Medicine, Imperial College London, London W12 0NN, UK; ^2^Department of Medicine, Duke University Medical Center, Durham, NC 27710, USA; ^3^Chronic Disease Research Centre, The University of the West Indies, Bridgetown 11115, Barbados

## Abstract

Cantharidin skin blisters were examined over two days to model the acute and resolving phases of inflammation in human skin. Four blisters were created by topical administration of cantharidin (0.1% v/v) to the forearm of healthy volunteers, with IRB approval. Duplicate skin blisters were aspirated at 16 and 40 hours to model the proinflammatory and resolving phases, respectively. There was a significant increase in leukocyte infiltrate at 40 h with appearance of a “resolving macrophage” phenotype CD14^+^CD163^+^ by flow cytometry. Neutrophils acquired apoptotic markers at 40 h and were observed to be phagocytosed by macrophagic “Reiter's” cells. Multiplex cytokine analysis demonstrated that monocyte chemoattractant protein (MCP-1/CCL2), interleukin- (IL-) 6, IL-8/CXCL8, macrophage inflammatory protein (MIP1**α**/CCL3), MIP-1**β**/CCL4, tumor necrosis factor- (TNF-) **α**, and eotaxin (CCL11) were all significantly upregulated at 16 h compared with 40 h. In contrast, immunoregulatory transforming growth factor- (TGF-) **β**, macrophage-derived chemokine (MDC/CCL22), and interferon-inducible protein (IP-10/CXCL10) were significantly elevated at 40 h. Our results demonstrate that the phases of inflammation and resolution can be discriminated in a two-day model of dermal wound healing. This confirms and extends our understanding of wound repair in humans and provides a powerful research tool for use in clinical settings and to track the molecular benefits of therapeutic intervention.

## 1. Introduction

The wound healing process is balanced between an early cytodestructive inflammatory phase and a subsequent resolving phase supporting tissue regeneration [1, 2]. Cells of the monocyte/macrophage lineage have been recognized since the 1970s to participate actively in both of these phases [3]. A key switching point is the conversion of the proinflammatory monocyte into a macrophage phenotype capable of dampening the inflammatory response and moulding fibrosis [4, 5]. The prototypic marker of this conversion is CD163 (the haemoglobin scavenger receptor), first recognised as the “resolving macrophage marker” in humans and as the ED2 antigen in rats [6, 7].

CD163 plays an everyday role in neutralising pro-oxidant free heme released during hemolysis in bruising or tissue injury. In a rat model of lung injury, it was expressed during inflammatory resolution at which macrophages engulfed apoptotic neutrophils [8]. In a model of gout involving monosodium urate crystal phagocytosis, the CD163^+^ phenotype evolves at the stage when monocytes/macrophages switch production from proinflammatory TNF*α*/IL-1/IL-6 cytokines to anti-inflammatory TGF-*β* [9]. In atherosclerotic plaques, CD163^+^ macrophages dampen oxidative injury due to intraplaque hemorrhage [10, 11]. The CD163^+^ phenotype is therefore a hallmark of the wound healing macrophage [12]. 

Despite a wealth of research in animal models, a molecular dissection of wound repair in humans and the role played by macrophage differentiation have been difficult to achieve due to lack of a convenient model.

The early inflammatory phase of wound repair is characterised by a predominantly granulocytic wave of leukocyte recruitment governed by neutrophil chemoattractants like Gro-*α* (CXCL1) and IL-8 (CXCL8) [13]. However, monocytes also participate in the first wave with IL-1, IL-6, TNF*α*, and MCP-1/CCL2 as prominent chemoattractants [13–16]. There remains some debate as to the importance of MIP-1*α*/CCL3 and MIP-1*β*/CCL4 in humans, but these mediators recruit leukocytes to wounds in animals [14, 17]. 

The blistering agent cantharidin has been in clinical use since the 1970s and is used as a topical treatment for molluscum contagiosum and warts. It is a protein phosphatase 1 and 2 alpha inhibitor [18]. When applied to skin it causes acantholysis and blister formation [19]. No serious adverse reaction for topical use of cantharidin has been reported in the literature [20]. The experimental use of cantharidin, as a model to study leukocyte trafficking, involves topical application of cantharidin at one seventh the clinical dose to the forearm, causing a blister of median volume 0.5 mL [21]. Blister fluid sampled between 16 hours and 24 hours exhibits the hallmarks of acute inflammation, with infiltration of inflammatory leukocytes and accretion of inflammatory cytokines, such as IL-8 and TNF*α*. In mice, an analogous model of inflammation has been developed using cantharidin in an ear swelling model [22]. We have further refined this technique to allow comprehensive analysis of the surface phenotype on blister emigrated leukocytes by flow cytometry [23].

Here, we have investigated whether cantharidin skin blisters can be extended into a second day to model the resolving phase of wound repair in human skin. We have compared chemokine and cytokine profiles in blister fluid at 16 hours versus 40 hours using multiplex technology and monitored conversion of the monocyte/macrophage lineage into the wound healing CD163 phenotype by flow cytometry.

## 2. Methods

### 2.1. Reagents

Cantharidin (Cantharone) was purchased from Dormer Laboratories Inc. (Rexdale, ON, Canada). Anti-CD163-FITC was purchased from Bachem (Merseyside, UK). Anti-CD14-ECD, control IgG-ECD, and control IgG-FITC were purchased from Beckman Coulter (High Wycombe, UK). Anti-CD16-FITC was purchased from Serotec (Kidlington, UK).

### 2.2. Human Subjects

Ten healthy human volunteers were enrolled into this study with informed consent. The study protocol was approved by the Hammersmith Hospitals Research Ethics Committee. One adverse event (hyperpigmentation that persisted after the blister had healed) was reported to the Research Ethics Committee, but the hyperpigmentation resolved eventually and enrolment was allowed to continue using a revised consent form. All human investigations were conducted according to the principles expressed in the Declaration of Helsinki.

### 2.3. Cantharidin-Induced Skin Blisters

A total of four skin blisters were created on the volar aspect of the forearm in each subject by topical application of Cantharone (Dormer Laboratories) at a concentration of 0.1% in acetone as described [23]. Two skin blisters were randomly assigned to the 16-hour timepoint and two skin blisters to the 40-hour timepoint. Blister fluid was collected into siliconized microcentrifuge tubes (Sigma Aldrich, Poole, UK) and stored on ice prior to cell counting and flow cytometric analysis. Total viable cell counts were determined using Trypan Blue stain, followed by counting in a hemocytometer. Differential cell counts were performed on a subset of 7 subjects using Kimura's stain, with counting in a hemocytometer. Blister supernatants were collected after microcentrifugation and stored for analysis of chemokines and cytokines at −70°C.

### 2.4. Flow Cytometric Analysis

Flow cytometric analysis of leukocytes was performed immediately after aspiration of blisters and microcentrifugation of samples, with all incubation and washing steps carried out in ice-cold PBS. CD163 expression on monocyte/macrophages was carried out in the fluorescent-(FL-)1 channel on cells gated with anti-CD14 antibody in the FL-3 channel. CD163 expression in whole blood was determined using the Immunolyse whole blood lysing technique (Beckman Coulter, Luton, UK.) as previously described. Apoptosis on the gated neutrophil population was carried out by two methods: measuring loss of expression of CD16 in the FL-1 channel or using the Annexin V-FITC Apoptosis Assay in the FL-1 and FL-2 channels as per manufacturer's recommendations (BD-Pharmingen, San Diego, CA). 

### 2.5. Multiplex Cytokine/Chemokine Analysis

The human cytokine multiplex-25 bead array kit was purchased from Biosource International (Camarillo, CA). The following cytokines/chemokines were screened: eotaxin (CCL11), granulocyte-macrophage colony-stimulating factor (GM-CSF), interleukin-(IL-)1*β*, IL-2, IL-2R, IL-4, IL-5, IL-6, IL-7, IL-8/CXCL8, IL-10, IL-12(p40/p70), IL-13, IL-15, IL-17, IL-1 receptor antagonist (IL-1RA), interferon-(IFN-)*α*, IFN-*γ*, interferon-*γ* inducing protein 10 kDa (IP-10/CXCL10), monocyte chemotactic protein (MCP-1/CCL2), macrophage inflammatory protein (MIP-1*α*/CCL3), MIP-1*β*/CCL4, regulated upon activation normal T cell expressed and secreted (RANTES/CCL5), monokine induced by *γ*-interferon (MIG/CXCL9), and tumor necrosis factor-(TNF-)*α*. Standard curves for each cytokine (in duplicate) were generated by using the reference cytokine concentrations supplied in this kit. Blister samples were diluted 2-fold in appropriate assay diluent. The assay was performed in a 96-well filter plate, using all the assay components provided in the kit. All incubation steps were performed at room temperature and in the dark to protect the beads from light. Samples were analysed using the Luminex 100 IS Multiplex Bio-Assay Analyzer (Bio-Rad Laboratories, Hercules, CA). 

### 2.6. Enzyme-Linked Immunosorbent Assays

Transforming growth factor-(TGF-) *β*, macrophage-derived chemokine (MDC/CCL22), IL-8/CXCL8, and MCP-1/CCL2 protein levels in blister fluid were measured in triplicate by capture ELISA (R&D Systems, Abingdon, UK) according to the manufacturer's recommendations. Cytokine/chemokine levels were expressed as mean concentration (pg/mL) ± SE.

### 2.7. Protein Membrane Array Assay

The RayBio cytokine membrane assay kit 5.1 allowed detection of 79 different human cytokines and chemokines from a single 1 mL fluid sample. Representative 16-and 40-hour blister supernatant samples were thawed slowly at 4°C and diluted 1 : 10 before performing the membrane array assay according to the manufacturer's instructions (RayBiotech Inc., Norcross, GA).

### 2.8. Statistical Analysis

Comparisons between groups were analysed using an unpaired Student's *t*-test. Statistical analysis was carried out using Graphpad Prism software (GraphPad Software Inc., La Jolla, CA), and significance was assumed at *P* < 0.05.

## 3. Results

### 3.1. Analysis of Leukocyte Infiltrates into Skin Blisters at 16 Hours and 40 Hours

**Figure 1:**
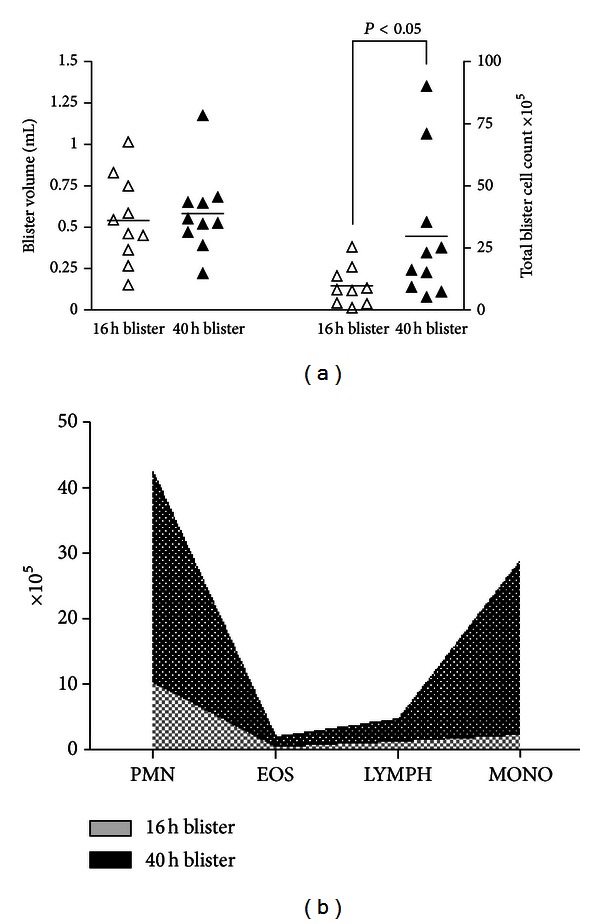
Leukocyte infiltration into cantharidin-induced skin blisters. Quadruplicate skin blisters were established by topical application of cantharidin (0.1%) to the forearm of 10 healthy individuals. Blister fluid was collected from duplicate blisters at 16 h and 40 h, respectively, and analyzed as follows (a) Fluid volume (mL) and cellularity (×10^5^). Each point represent the average from 2 skin blisters; horizontal lines represents the population mean. (b) Differential leukocyte subpopulations. PMN: polymorphonuclear phagocytes; EOS: eosinophils; LYMPH: lymphocytes; MONO: monocytes/macrophages.

There was a statistically significant increase in leukocytes infiltrating into skin blisters at 40 hours compared to 16 hours (*P* < 0.05; Figure 1(a)). However, the volume of blister fluid did not alter significantly (Figure 1(a)). Blister cellularity was not correlated with blister volume (*r* ^2^ = 0.03; *P* = 0.54). Analyzing the leukocyte subpopulations within blister fluid revealed a marked increase in the number of neutrophils and monocytes/macrophages per blister present at the 40-hour timepoint (Figure 1(b)). The proportion of neutrophils, monocytes/macrophages, lymphocytes, and eosinophils present in blister fluid is illustrated in Table 1.

**Figure 2:**
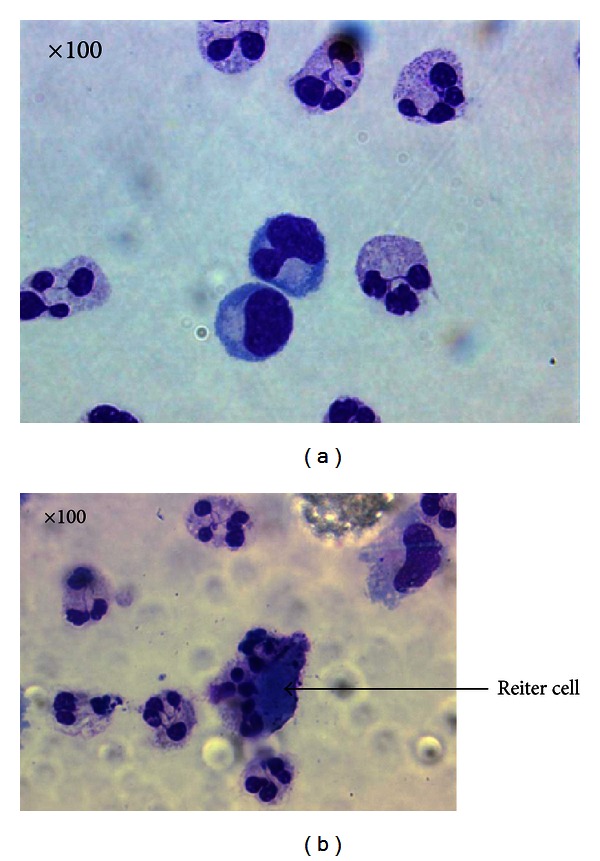
Photomicrographs of blister fluid at (a) 16 hours and (b) 40 hours. Granulocytes reveal morphological changes characteristic of apoptosis at 40 hours, including vacuolation of cytoplasm, size shrinkage, and engulfment by phagocytic “Reiter's” cells (arrow). Magnification = 100x.

The morphological appearance of neutrophils by light microcpopy altered over time, consistent with apoptosis. Vacuolated cytoplasm, condensation, and cell membrane degradation were observed at 40 h (Figure 2(b)). Also detected in cytospin, preparations of blister fluid at 40 hours were large macrophages that had engulfed apoptotic neutrophils (“Reiter's” cells).

### 3.2. Flow Cytometric Determination of Neutrophil Phenotype

**Figure 3:**
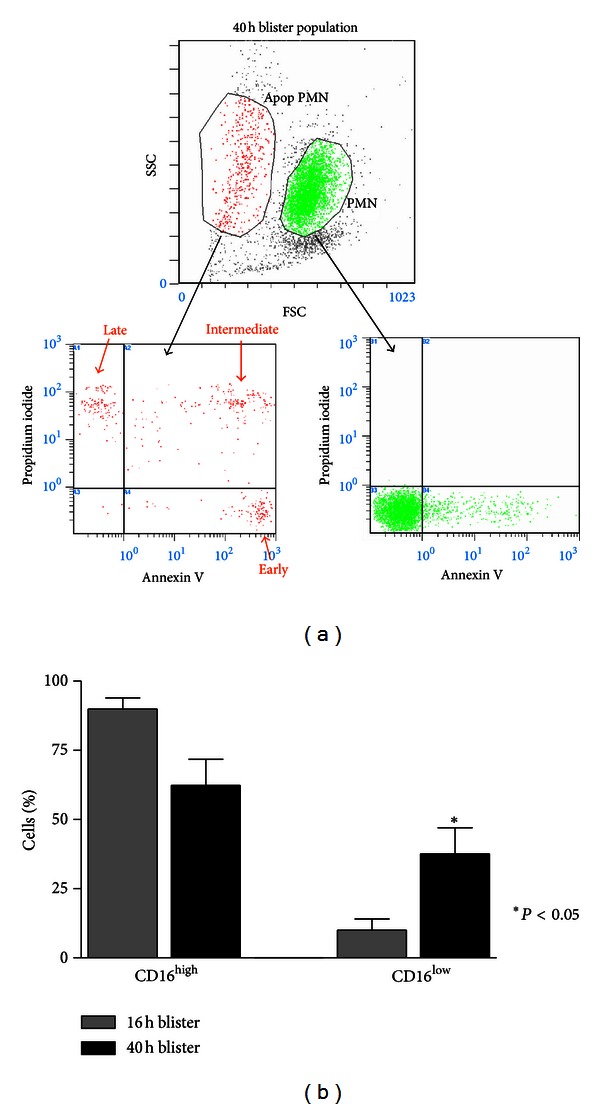
Flow cytometric analysis of PMNs in blister fluid. (a) Forward and side-scatter analyses reveal a subpopulation of smaller, less granular PMN. Gating on this subpopulation demonstrates expression of apoptosis markers, Annexin V, and Propidium Iodide (PI). Annexin V^+^ cells are considered at an early stage of apoptosis, Annexin V^+^/PI^+^ cells are at an intermediate stage, and Annexin V^−^/PI^+^ cells at a late stage of apoptosis. No expression of apoptosis markers is seen in the viable cell gate. (b) PMNs at 40 hours demonstrate an increased proportion of cells exhibiting a CD16^low^ phenotype, also characteristic of apoptotic cells.

Forward and side-scatter profiles of the granulocyte gated population in blister fluid revealed a distinct smaller, less granular sub-population at 40 hours (Figure 3(a)). By placing a gate over this new cell population, these were shown by Annexin V and Propidium Iodide staining to contain apoptotic and necrotic cells (Figure 3(a)). These cells also exhibited diminished expression of CD16, a characteristic of neutrophil apoptosis [24]. Whereas CD16^low^ cells comprised 10.05% ± 9.72 (mean ± S.D.) of the population at 16 hours, this rose to 37.6% ± 28.3 at 40 hours (*P* < 0.05) (Figure 3(b)). Hence, flow cytometric data for apoptosis markers supported the observations of light microscopy showing an increase in apoptotic neutrophils at 40 hours in the blister transudate. 

The purity of gated leukocyte sub-populations was verified by CD16/VLA-4 double-staining in the granulocyte and mononuclear cell gates. The percentage of CD16^−^VLA-4^+^ (monocytic cells) cells contaminating the granulocyte gate was 2.2% ±  0.7 (mean ± SEM, *n* = 9), and likewise the percentage of CD16^+^VLA4^−^ (neutrophilic) cells in the mononuclear gate was 5.0% ± 0.8, confirming that the gating strategy based on forward and side-scatter profiles combined with CD14^+^ marker was specific enough to discriminate between neutrophil and monocyte/macrophage lineage populations.

### 3.3. Flow Cytometric Determination of Monocyte/Macrophage Phenotype

CD163 is a monocyte-macrophage lineage marker expressed by alternatively-activated macrophages during the resolving phase of inflammation [7, 25]. The monocyte/macrophage population at 40 hours shows clear evidence for differentiation into an alternatively activated end-point, with a significant increase (*P* < 0.01) in the proportion of CD14^+^CD163^+^ double positive cells (47.6% ± 7.6 at 40 h (mean ± S.D.) compared to 3.4% ± 1.1 at 16 h and 4.0% ± 1.1 within the circulation). This adds to the evidence that by 40 hours the cellular infiltrate within the blister reflects a resolving macrophage phenotype.

### 3.4. Chemokine and Cytokine Expression in Blister Supernatant

**Figure 4:**
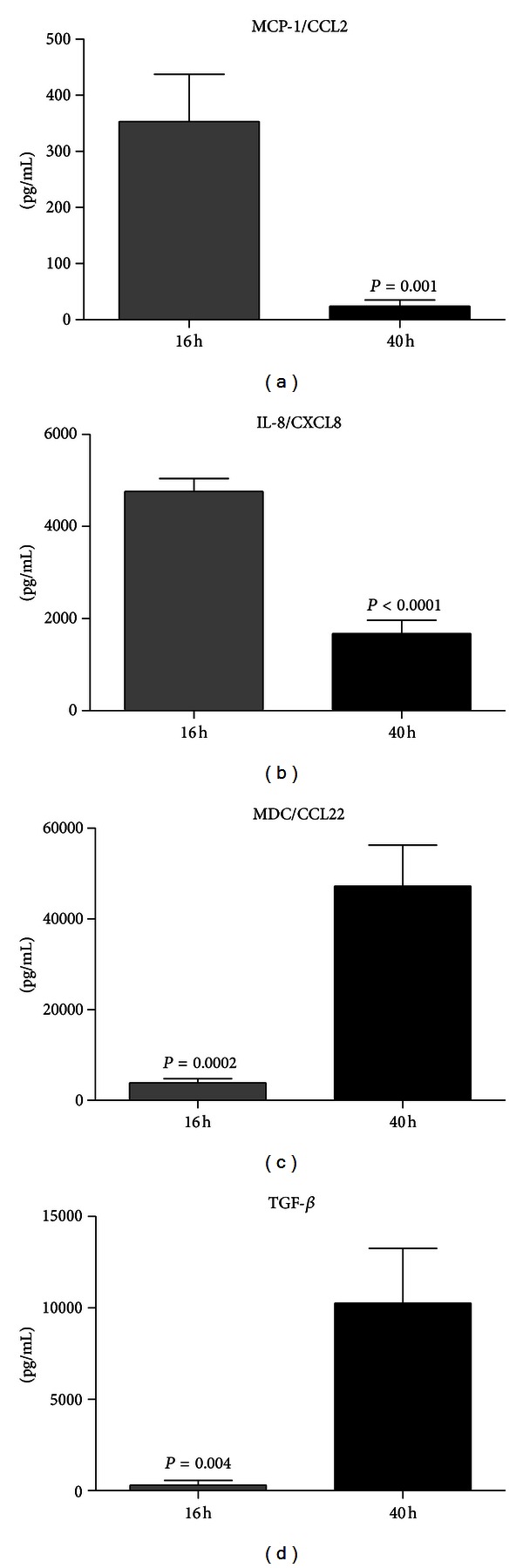
Enzyme-linked immunosorbent assay (ELISA) detection of inflammatory regulators in blister fluid: (a) MCP-1/CCL2, (b) IL-8/CXCL8, (c) MDC/CCL22, and (d) TGF*β*. Blister fluid from *n* = 10 individuals was analyzed by ELISA in duplicate, at each of the timepoints. Results are expressed as mean ± S.E.M. *P* values represent statistical differences between 16-hour and 40-hour timepoints analyzed by unpaired *t* test.

To examine the soluble inflammatory mediators within blister supernatants, a cytokine array was chosen as an initial screening step prior to quantitative analysis by ELISA. The array could detect up to 79 different cytokines using only 1 mL of blister sample fluid. Blister supernatant from a randomly selected individual was diluted 1 : 10 and run on the array. The results pointed towards a changing inflammatory status within the blister over time (data not shown). The 16 h blister supernatant was strongly positive for proinflammatory cytokines IL-8/CXCL8 and MCP-1/CCL2, but by 40 h these cytokines had decreased. A weak signal for MDC/CCL22 was detected at 40 hours and submitted for confirmatory testing by ELISA. ELISA confirmed the observations of the protein array (Figures 4(a)–4(c)), with IL-8/CXCL8 and MCP-1/CCL2 significantly elevated at 16 hours, but MDC/CCL22 exhibiting an opposite profile (i.e., higher at 40 hours). 

**Figure 5:**
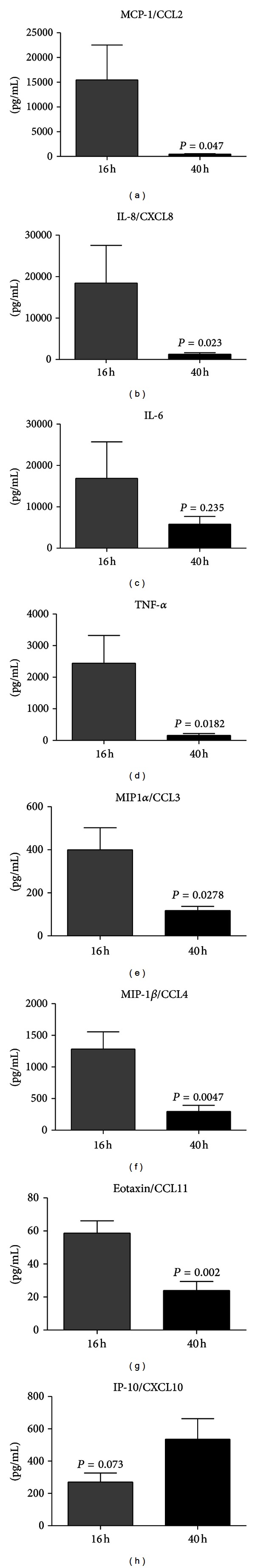
Multiplex bead array (Luminex) analysis of inflammatory regulators in blister fluid: (a) MCP-1/CCL2, (b) IL-8/CXCL8, (c) IL-6, (d) TNF*α*, (e) MIP-1*α*/CCL3, (f) MIP-1*β*/CCL4, (g) Eotaxin/CCL11, and (h) IP-10/CXCL10. Blister fluid from *n* = 10 individuals was analyzed by multiplex bead array at each of the timepoints. Results are expressed as mean ± S.D. *P* values represent statistical differences between 16-hour and 40-hour timepoints analyzed by unpaired *t* test.

Next, blister fluid from the complete study panel (*n* = 10) was analysed by multiplex bead array for the presence of 25 human cytokines, chemokines, and growth factors. The results of this analysis confirmed the previous observations for IL-8 and MCP-1 by protein array and ELISA, showing a statistically significant increase at 16 h compared to 40 h (Figures 5(a) and 5(b); *P* < 0.05). Similar patterns were also observed for five other pro-inflammatory mediators, namely, TNF*α*, IL-6, MIP-1*α*, MIP-1*β*, and eotaxin (Figures 5(c)–5(g); all statistically significant *P* < 0.03, except for IL-6 trend, *P* = 0.235). The opposite pattern was observed for IP-10, which was elevated at 40 h compared to 16 h (Figure 5(h); statistical trend *P* = 0.073). There were no statistically significant changes or trends in expression for the following cytokines/chemokines/growth factors: IL-1*β*, IL-1RA, IL-2, IL-2R, IL-4, IL-5, IL-7, IL-10, IL-12(p40/p70), IL-13, IL-15, IL-17, GM-CSF, IFN-*α*, IFN-*γ*, IP-10/CXCL10, MIG/CXCL9, and RANTES/CCL5. Finally, the important wound healing cytokine TGF-*β*, which was not represented on the bead array, was shown by ELISA to have the same expression pattern as MDC/CCL22 and IP-10/CXCL10 (i.e., elevated at 40 hours compared to 16 h hours) (Figure 4(d); *P* = 0.004).

## 4. Discussion

The present study establishes cantharidin skin blisters as a tool for tracking the two main phases of wound healing in humans. By extending blisters into a second day and by analyzing blister infiltrates using flow cytometry and multiplex cytokine arrays, we were able to detect the following hallmarks in the transition from a pro-inflammatory phase at 16 h to a resolving state at 40 h: (1) a switch from proinflammatory mediators (IL-6, IL-8/CXCL8, MCP-1/CCL2, MIP-1*α*/CCL3, MIP-1*β*/CCL4, TNF*α*, and eotaxin/CCL11) to immunoregulatory chemokines and growth factors (TGF-*β*, MDC/CCL22, and IP-10/CXCL10), (2) transition of CD14^+^CD163^−^ monocytes to a resolving macrophage CD14^+^CD163^+^ phenotype, and (3) acquisition of apoptosis markers by neutrophils and evidence for their phagocytosis by Reiter's cells. 

The cantharidin blister model has enabled a molecular dissection of the wound healing process in humans, which has confirmed and extended our existing understanding of dermal wound repair in humans. Our data confirms the importance of IL-6, IL-8/CXCL8, MCP-1/CCL2, MIP-1*α*, MIP-1*β*, and TNF*α* in the early inflammatory phase [13, 14, 16, 26, 27]. New insights were gained into eosinophil recruitment into skin, with eotaxin exhibiting an early expression profile similar to MCP-1/CCL2 and IL-8/CXCL8. This is consistent with a prominent role for this cell type in the wound repair process, as proposed previously [28, 29]. The sequence by which leukocytes were recruited into blisters was broadly similar to that previously described in an incisional wound model in humans, with neutrophils predominating at the early timepoint followed by a wave of monocyte/macrophage recruitment [13]. Neutrophil apoptosis and phagocytosis by macrophagic Reiter's cells were also captured in second day blisters. Whereas one-day-old cantharidin blisters have been previously used to demonstrate impaired neutrophil migration in Crohn's disease [30], extending blisters into a second day might enable impaired clearance of apoptotic cells to be studied in autoimmune diseases. Lymphocytes accumulated at 40 h, but it was not possible to examine later timepoints, since blisters became too fragile. It was notable that T cell chemoattractants implicated in wound repair [13], like MIG/CXCL9, and T cell cytokines, such as interferon-*γ*, were absent, but again it was not possible to examine any later timepoints in this study.

One of the most striking features of our data was the rapidity with which proinflammatory mediators were lost in the intervening 24 hours between the first and second sampling points. Bearing in mind that we studied an accumulation model, one would have expected cytokines present at 16 h to be still there at 40 h, unless they had been actively broken down, quenched, or extruded from the blister. We observed that concentrations of MCP-1/CCL2, IL-8/CXCL8, and TNF*α* each fell >1 log between 16 h and 40 h. The rapid loss of inflammatory mediators is consistent with the possibility of proteolytic degradation or quenching by proteoglycans, which has been described for chemokines [31–33], but either of these options remains to be proven. There was also a rapid switch to immunoregulatory factors in the blister model, with high levels of TGF*β* detected at 40 h. This is quicker than has been reported previously using full thickness wound models where TGF*β* peaks at 7 days [34], but the speedier transition to the resolution phase may have been be due to the fact that cantharidin skin blisters do not penetrate the dermis, obviating the need for a prolonged inflammatory or granulation step. The two-day blister model may be well suited for examining impaired wound healing in diabetes, where TNF*α* dysregulation has been shown to drive inflammatory and apoptotic processes, as well as impairing signalling in wounds [35, 36]. 

There was a marked increase in cells of the monocyte/macrophage lineage at the 40 h timepoint, and CD163^+^ macrophages accounted for almost 50% of this cell population. The conversion into the “resolving macrophage” phenotype was quicker than previously reported in a subcutaneous rat model, which required 21 days to achieve a similar 43%–56% conversion into the ED2 phenotype [37]. Again, the likely explanation lies with the fact that the skin blister model provides a less complicated lesion than the skin implant model studied in rats and the transition to the resolution phase is quicker. Previous work has revealed an important role for free haemoglobin driving CD163 expression via an IL-10 feedback loop [10]. However, this mechanism does not appear to operate in skin blisters since there was no hemorrhagic component detected in skin blisters and IL-10 was undetectable at either timepoint [38]. The lack of IL-10, IL-4, and IL-13 detected in our model is consistent with the same observation made in animal wounds [39]. The identity of the polarising factor for CD163 conversion in skin is the aim of ongoing investigation, since CD163 expression is linked to beneficial antioxidant and anti-inflammatory pathways in macrophages [7, 10, 12, 40–43]. 

While cantharidin skin blisters present fewer ethical implications for wound research in humans than incisional or excisional models of wound healing, they also present some limitations. The rapidity of proinflammatory cytokine switching and shift to the CD163^+^ phenotype is clearly at odds with past research in full thickness injury models which exhibited a slower timecourse. Hence, it is unclear whether the molecular details of wound repair learned in the skin blister model can be extended to deeper wounds. Nonetheless, neutrophil apoptosis and phagocytic clearance by macrophagic cells with accompanying TGF*β* release were reproduced in the skin blister model, suggesting that the major hallmarks of inflammatory resolution were present. 

In conclusion, we have demonstrated that skin blisters extended into a second day reproducing the major features of the resolving phase in wound healing. The usefulness and safety of using cantharidin skin blisters have already been demonstrated for discriminating immediate and delayed inflammatory responders and in studies of the systemic inflammatory response to cardiopulmonary bypass and patients with inflammatory diseases, such as Crohn's [27, 30, 44]. Extension of the cantharidin skin test into the second day may find therapeutic application in evaluating wound healing treatments or proresolving anti-inflammatory interventions [45]. 

## Figures and Tables

**Table 1:** Proportion of leukocyte subsets in skin blisters.

	Neutrophils	Eosinophils	Lymphocytes	Monocytes
16 h blister	68.9%	6.6%	7.7%	16.8%
40 h blister	56.5%	6.0%	7.9%	29.7%
